# Reestablishment of native top‐predator suppresses invasive competitor in coastal Norway

**DOI:** 10.1002/ecy.70597

**Published:** 2026-11-13

**Authors:** Steven Guidos, Øystein Varpe, Jiska van Dijk, Arild Landa

**Affiliations:** ^1^ The Norwegian Institute for Nature Research Bergen Norway; ^2^ Department of Biological Sciences The University of Bergen Bergen Norway; ^3^ The Norwegian Institute for Nature Research Lillehammer Norway

**Keywords:** camera trapping, competition, exclusion, invasive species, *Lutra lutra*, *Neogale vison*

## Abstract

Invasive predators are among the leading drivers of biodiversity loss worldwide, but their impacts may be mitigated by native intraguild competitors. The recovery of the Eurasian otter (*Lutra lutra*) in Europe has laid the foundation for numerous studies on the competitive effects of a dominant native predator on an invasive inferior, the American mink (*Neogale vison*). Here, we exploited the otter's natural expansion direction along the coast of Norway to establish two study areas to test whether the otter's level of reestablishment influenced mink activity, spatial occupancy, and behavior via camera trapping. Mink site occupancy was significantly lower in the area where otters have reestablished (established region), and daily detection rates were 72% lower than in the area with only transient otters (expansion region). We found no evidence of day‐night shifts in mink diel activity patterns in response to otters. However, mink in the established region displayed significantly more investigatory behavior, which declined in response to increased time since the last otter detection. There was substantial overlap in preferred daily weather conditions for both species, which could lead to increased encounter rates and is a possible explanation for decreased mink detections where they overlap with otters. Our study supports that native apex carnivores can exert top‐down control on invasive competitors, and we discuss the broader implications of this finding in light of global management challenges. We recommend further integration of GPS telemetry with camera trapping to assess fine‐scale spatial interactions between the study species and to allow for robust density estimates between regions.

## INTRODUCTION

Invasive species are one of the leading causes of species extinctions and biodiversity loss worldwide (Mollot et al., 2017; Pyšek et al., 2020). Once established, invasive species are generally free from their natural competitors, predators and parasites (Keane & Crawley, 2002), and many possess traits like high fecundity and growth rate (Jeschke & Strayer, 2006), aiding expansion. Few ecosystems have evaded their impacts, and in cases where the invasive species is a mammalian predator, they can be particularly damaging to insular ecosystems, where prey species are naïve to predation (Bellard et al., 2016; Medina et al., 2011; Szabo et al., 2012). Doherty et al. (2016) implicated invasive mammalian predators in the extinctions of at least 87 bird, 45 mammal, and 10 reptile species globally.

However, damaging impacts of invasive predators can potentially be reduced in ecosystems where there are already intraguild competitors (Cunningham et al., 2020; Gordon et al., 2015; Rivera‐Estay et al., 2024). Establishment success is therefore often related to extent of niche overlap with native species, adaptability, and availability of resources (Brzeziński et al., 2021). For invasive predators, it has been shown that establishment and coexistence with native competitors can theoretically be promoted when the invasive is a generalist, capable of preying upon a large assortment of species, and the native is a specialist with a smaller niche breadth than the invader (Bonesi et al., 2004).

One such generalist‐specialist relationship is that of the American mink (*Neogale vison*, hereafter “mink”) and the Eurasian otter (*Lutra lutra*, hereafter “otter”) in Europe. Following importation of mink for fur farming, escapees established throughout much of Europe in the mid‐20th century (Dunstone, 1993), a time in which most otter populations in Europe were displaced from a combination of hunting and habitat degradation (Mason & Macdonald, 1994). Mink predate heavily on European ground‐nesting seabirds and riparian birds (Brzeziński et al., 2018; Craik, 1995; Ferreras & Macdonald, 1999; Jónsson et al., 2023; Nordström et al., 2003), and they have received much of the blame for the dramatic decline of water voles (*Arvicola amphibius*) in Britain (Barreto et al., 1998; Woodroffe et al., 1990). Mink have also been linked to the decline of a closely related competitor, the European mink (*Mustela lutreola*) (Maran & Henttonen, 1995; Põdra et al., 2013). After receiving protection during the 1980s, the otter started to reestablish in parts of Europe, which led to questions about what impact this would have on the invasive mink population, given the potential niche overlap of the species.

The competitive relationship between the two species is a topic of debate, and many studies have tried to decipher potential spatial and temporal displacement of the otter on mink (Bonesi et al., 2006; Bonesi & Macdonald, 2004a, 2004c; Harrington, Harrington, & Macdonald, 2009; Harrington, Harrington, Yamaguchi, et al., 2009), while others have attempted to understand dietary competition (Bueno, 1996; Clode & Macdonald, 1995; Harper et al., 2020). Some studies have indicated that mink occupancy of river systems drops following otter reestablishment (Bonesi & Macdonald, 2004c), others document rhythmic activity pattern shifts by mink to avoid temporal overlap with otters (Harrington, Harrington, Yamaguchi, et al., 2009). Several studies point to mink dietary shifts in the presence of otters to avoid competition over food resources (Bueno, 1996; Clode & Macdonald, 1995). In general, it is believed that ability to coexist will be dependent on habitat heterogeneity and ability to reduce niche overlap by mink (Bonesi & Macdonald, 2004a).

However, many of the past investigations on this competitive relationship have relied on mink and otter scat detection between different study regions or over time. Recent studies have criticized the use of scat detection as a method (Harrington et al., 2020), given that rates of scat deposition can change based on environmental factors or in response to otter presence, even if mink activity levels remain stable (Bonesi & Macdonald, 2004b; Harrington & Macdonald, 2015). Even experienced surveyors can have trouble distinguishing between mink, otter, and polecat (*Mustela putorius*) feces in areas where they are sympatric (Harrington et al., 2010). In addition, camera trapping has indicated that close to half of otter observations would be unaccounted for should the observer rely on scat detection alone (Guter et al., 2008). If the same is true of mink, this would throw into question the validity of results found in earlier scat‐based studies. As such, there is need to reevaluate past conclusions with updated methods.

Mink were introduced to Norway in 1927 for fur farming (Bevanger & Henriksen, 1995). At this time, the Norwegian otter population was widely persecuted, and remnant populations were relegated to northern parts of the country and Hedmark County (Christensen, 1995). Within a 35 year period, mink established 80%–85% of the Norwegian mainland (Bevanger & Henriksen, 1995) free from otter competition. Following nationwide protection in 1982, the otter managed to reestablish much of its previous historical range, and the current area of expansion is in Rogaland County and continuing southward (Landa & Guidos, 2020). A secondary expansion is also coming westward from Innland County and Sweden (van Dijk et al., 2020). As such, the otter now cohabits much of Norway with the mink. However, competition between the two species in Norway has not been properly investigated, and studies in coastal, marine environments are lacking compared to limnic systems.

In this study, we exploited the otter's natural reestablishment southward along the western coast of Norway to compare areas of varying otter inhabitance, in which we could study mink activity patterns in regions which are at the forefront of the otter reestablishment zone, and compare to areas where they have cohabited for over a decade. To avoid known biases associated with scat collection, we used camera traps to monitor site occupancy and rhythmic activity between study regions. The use of camera traps also allowed for documentation of other valuable information such as prey items, litter sizes, and behavior.

We predicted lower site occupancy and detection frequency on camera traps in the established area (mink sympatric with otters), compared to the expansion area (mink populations generally free from otter competition). We also predicted that mink detection frequency would be less at camera stations where otters had recently been. In addition, we predicted that mink would be more diurnal in the established area compared to otters, which are expected to be mostly active between dusk and dawn. We also hypothesized that mink behaviors related to site “investigation” would be influenced by time since the last otter observation. Lastly, we tested if detection frequencies were influenced by environmental variables such as cover type and amount, and daily weather patterns.

## METHODS

### Study region

The established region is located in Vestland County (Figure 1), and the first recorded otter observation in the area was in 2012 (Artsdatabanken.no, 2025). Otters and mink have thus been coexisting in the established region for at least 11 years prior to the start of the study. The expansion region is in Agder County, which currently lies at the forefront of recent otter reestablishment in Norway. The mink population in this region is considered to exist free from otter competition, as recent otter sightings on public databases (Artsdatabanken.no, 2025) in the area have been infrequent and are likely transient individuals.

**FIGURE 1:**
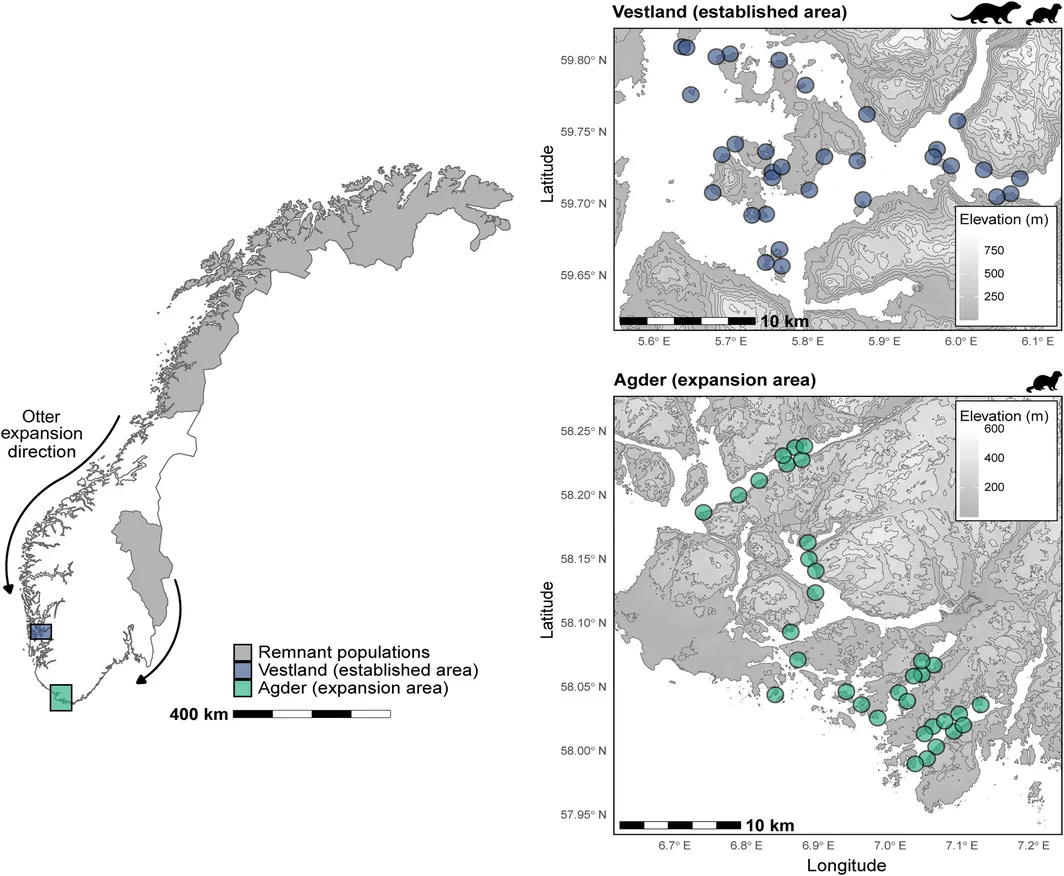
An overview of the reestablishment direction of the Eurasian otter southward along the coast of Norway in relation to the locations of the expansion and established areas (left). Elevation maps (right) of both study regions, along with respective camera placements. Otter and mink silhouettes were created using generative software (OpenAI, ChatGPT).

The expansion region was chosen based on its ecological similarity to the established region. Both are composed of fjord systems, with narrow saltwater inlets which quickly ascend to steep, mountainous terrain. Shorelines are generally narrow but boast dense macroalgal growth (mostly fucoids) in the littoral zone (Husa et al., 2014). Common marine prey species in both regions belong to the Gadidae, Labridae, Gobiidae, Galatheidae, and Carcinidae families (Salvanes & Nordeide, 1993). Small streams are common in both areas but are usually nutrient poor given the short distances from source to mouth and are prone to drying in periods of low rainfall. Freshwater prey resources suitable for mink are therefore limited compared to along the shoreline. Terrestrial prey species include wood mice (*Apodemus sylvaticus*) shrews (*Sorex* spp.) and a diverse assemblage of passerines, waders, and shorebirds. Competitors like red foxes (*Vulpes vulpes*), pine martens (*Martes martes*), and stoats (*Mustela erminea*) are common in both study areas (Artsdatabanken.no, 2025).

As mink is an invasive species, it is possible to trap them without a license. Outside of important seabird colonies, mink trapping in Norway occurs on an uncoordinated, recreational basis. Spatial resolution of trapping records is too low (municipal level) to accurately estimate direct human impacts on the mink populations in each study area. However, given that the fjord regions studied here are sparsely populated, we assumed trapping effort to be low and not differ significantly between regions.

### Study design

Between March 1, 2023 and February 28, 2024, a total of 32 cameras were deployed in the established area, and 34 in the expansion region. Locations with potential mink activity were located by patrolling the shoreline by boat, and looking for latrine sites (which when placed on vegetation turn the immediate area a vibrant green), or by looking for piles of large boulders placed along the shoreline, which mink tend to frequent and collect scat over time. After locating probable areas of mink activity, the site was checked on foot for signs of mink such as scats, trails through vegetation or discarded remains of prey. Cameras were placed near mink trails within ca. 15 m of the shoreline, as mink activity tends to be concentrated near the coastal strip (Bonesi et al., 2000). In an effort to standardize camera placement relative to apparent mink activity levels, field teams aimed to place subsequent cameras at least 1 km from the nearest location at the next sign of mink presence. Mean distance between camera traps (by way of shoreline) was 2.2 km (SD = 1.1), which exceeds the known territorial range of female mink in coastal environments, but not males (1.2–1.4 km and 2.2–2.5 km for females and males, respectively [Bonesi, 1996; Ireland, 1990]). Most cameras therefore likely collected data from distinct female ranges, though male ranges may have overlapped with several camera traps.

All cameras were placed on metal tripods and bolted to exposed rock ca. 20 cm above ground, facing the direction of a trail. The cameras were the passive, infrared Reconyx Hyperfire 2 (Reconyx Inc., Wisconsin, USA). All were set to ¼ s trigger speed on “High” sensitivity, three pictures were taken per motion sequence, separated by 1 s. The cameras had no quiet period between detections. While all camera locations were assumed to be in equally suitable mink habitat, differences in cover amount (“none,” “partial,” and “total,”) and cover type (“none,” “rock,” “vegetation”) were noted to control for any potential variation in local habitat between cameras. By definition, “total” coverage refers to any kind of cover that would completely obstruct view of the mink and trail area from above. This includes trails within areas of dense scrub growth, wooded areas and large boulders. “Partial” cover refers to an area in which a mink would be visible from above when viewed at an angle. Such instances often occurred when trails ran next to overhanging cliff faces or alongside undergrowth. Proportion of each habitat variable was similar between the expansion and established area (see Appendix S1: Table S1 for a detailed overview of local habitat variation between cameras). Cameras were checked once every other month to change SD cards and batteries.

Annotation of images was done using the Agouti software (Agouti, 2025), which grouped pictures within 120 s of the first timestamp into sequences. Animal behavior in each sequence was assigned to one or several of the following categories: “Eating,” “Fighting,” “Foraging,” “Investigating,” “Mating,” “Playing,” “Resting,” “Scent marking,” and “Traveling.” Descriptions of each behavior can be found in Table 1. Sex of otters and mink were recorded when possible, along with presence of pups and litter sizes. Litter size was equal to the maximum number of individuals present in the same frame per sequence −1 (excluded the mother). Any observations of mink carrying prey items were recorded to species group when possible, otherwise as prey type.

**TABLE 1:** All behaviors recorded and their definitions.

Behavior	Definition
Eating	Individual has a parcel of food in its mouth.
Fighting	Individual is grappling/biting another. Clearly not playful.
Foraging	Individual is looking for food, picking at vegetation, and so forth (usually only applied to birds and rodents).
Investigating	Individual is moving slowly, usually with head to the ground, sniffing behavior.
Mating	Individual is copulating with another.
Playing	Individual is grappling/biting another, clearly not aggressive. Usually otter pups and/or mother.
Resting	Individual is loafing around or sleeping.
Scent marking	Individual is defecating or rubbing fur into grass.
Traveling	Individual travels past the camera at a constant rate, little or no change in direction.

### Statistics

Activity levels were standardized temporally by filtering observations that occurred within 1 h of the last recorded observation of the same species. Mink spatial use was evaluated using two metrics: (1) regional daily naïve occupancy, defined as the proportion of active camera stations that detected mink per date, and (2) daily detection frequency per camera. We used regional naïve occupancy to provide a broad daily index of mink occurrence across each study region, while detection frequency was modeled at the camera‐day level to evaluate ecological covariates and account for repeated observations at individual camera stations.

To assess spatial and temporal variation in daily occupancy between regions, we modeled naïve daily mink occupancy using generalized linear mixed‐effects models (GLMMs). For each date, naïve occupancy was defined as the number of camera stations that detected mink out of the total number of active camera stations per region, forming a binomial response specified as counts of successes and failures. As this metric does not account for imperfect detection, it is interpreted as daily camera detection incidence rather than true occupancy. A binomial GLMM with a logit link was used to account for varying sampling effort across dates and to estimate daily use probability. Preliminary assessment indicated substantial day‐to‐day autocorrelation and shared environmental conditions among cameras operating on the same date. Sampling date was therefore included as a random intercept to account for nonindependence among observations within days. Region and Month (treated as a categorical seasonal factor) were included as fixed effects, along with their interaction to allow region‐specific seasonal patterns in occupancy. The significance of fixed effects was evaluated using Wald Type II χ^2^ tests. Model assumptions were assessed using residual diagnostics, fitted‐versus‐observed plots, and inspection of random‐effect variance. Pairwise comparisons among months within regions were conducted using Tukey‐adjusted contrasts on the logit scale with the R package emmeans (Lenth, 2025).

Mink detection frequency, defined as the number of independent detections per camera per day, was modeled using GLMMs. Daily detection counts contained many zero values and were strongly right‐skewed and overdispersed; therefore, models were fitted using a negative binomial error distribution with a log link. Camera station and sampling date were included as random intercepts to account for repeated measurements at individual cameras and shared environmental conditions across cameras operating on the same day. Region and Month were included as fixed effects, along with their interaction. To assess whether mink detection frequency was influenced by the recency of otter presence at a camera, we calculated the number of hours elapsed since the most recent otter detection at each camera per date. Time since otter detection was treated as a continuous covariate. Potential nonlinear effects were modeled using natural cubic splines (3 df), evaluated as an additional fixed effect. Camera‐days with no previous otter detections could not be assigned a finite time‐since‐otter value; these observations were therefore retained in the model using a separate binary indicator for no prior otter detection, while the spline term was applied only to camera‐days with a finite time since previous otter detection. The impacts of long‐term camera‐level otter detection rate on mink detection rate was also assessed by taking the total number of independent otter detections divided by the number of active camera‐days and standardized. Daily temperature and precipitation were obtained from regional meteorological stations and matched to camera‐days by date and region. Wind conditions were derived using gridded atmospheric reanalysis data from the Copernicus Climate Data Store (2025) from which daily mean wind speed was extracted for each camera. Wave height data were not available for either study region; wind speed was therefore used as a proxy for coastal exposure. Alternative model formulations, including environmental covariates (cover amount, cover type, precipitation, temperature, wind speed, recency of otter presence, annual otter detection rate) with and without interactions with Region and Month, were evaluated sequentially using likelihood ratio tests and differences in Akaike's information criterion (AIC). Covariates that did not improve model fit were excluded from the final model. The significance of fixed effects in the retained model was assessed using Wald Type II χ^2^ tests. Tukey‐adjusted pairwise comparisons were used to evaluate differences among months within regions. The final GLMM for mink, excluding nonsignificant covariates was as follows:

$$\log\left(\mu \mathrm{ij}\;\right)=\beta_{0}+\beta_{1}\;{\text{Region}}_{i}+\beta_{2}\;{\text{Month}}_{j}+\beta_{3}\;\left({\text{Region}}_{i}\times {\text{Month}}_{j}\;\right)+\beta_{4}{\text{NoPriorOtter}}_{\mathrm{ij}}+f\left({\text{OtterRecency}}_{\mathrm{ij}}\right)\times \left(1-{\text{NoPriorOtter}}_{\mathrm{ij}}\;\right)+\beta_{5}\;{\text{WindSpeed}}_{\mathrm{ij}}+b_{i}+d_{j},$$

where μij is the expected number of mink detections at camera i on day j; Region and Month are fixed effects describing spatial and seasonal variation, including their interaction; ${\text{NoPriorOtter}}_{\mathrm{ij}}$ is a binary indicator identifying camera‐days for which no previous otter detection was available; $f\left({\text{OtterRecency}}_{\mathrm{ij}}\right)$ is a natural cubic spline (3 df) modeling the nonlinear effect of hours since the last otter detection at a camera; and multiplication by $\;\left(1-{\text{NoPriorOtter}}_{\mathrm{ij}}\;\right)$ restricts the spline effect to camera‐days with a finite time since previous otter detection. WindSpeed is mean daily wind speed (standardized); and $b_{i}$ and $d_{j}$ are random intercepts for camera station and sampling date, respectively, accounting for repeated measurements and shared daily conditions. Otter occupancy and detection frequency were analyzed using the same modeling framework as described above for mink, excluding otter recency and the no‐prior‐otter term as predictors.

To investigate potential differences in rhythmic activity patterns between regions, we classified each detection event into one of four activity periods: “Dawn,” “Day,” “Dusk,” and “Night.” Local sunrise and sunset data were based on camera coordinates and date using the suncalc package in R (Thieurmel & Elmarhraoui, 2017). Dawn and dusk were defined as the periods between astronomical twilight and sunrise, and between sunset and astronomical twilight, respectively. Daytime comprised the period between sunrise and sunset, excluding dawn and dusk, and night included all times outside these intervals. We summarized activity levels for each region by calculating the proportion of detections falling into each activity period. To test if the probability of daytime activity (i.e., detections classified as Day) varied between regions and months, we constructed a GLMM with a binary response variable (1 = Day, 0 = other) and included Region, Month, and their interaction as fixed effects. A random intercept for camera ID was included to account for repeated sampling across locations. Comparison of monthly activity patterns within regions was done using Tukey‐adjusted pairwise comparisons.

Mink observations were not filtered temporally when assessing differences in behavior between regions and in relation to otter occupancy. This was because behavior varied widely between observations that happened in quick succession, in which case a temporal filter would remove observations that were considered equally relevant. Individual detections often included multiple behaviors. The data were formatted prior to analysis so that each separate behavior noted per sequence was given a unique row. Total number of observations of behavioral traits was therefore greater than number of unique observations of mink and otters. Behaviors that were observed in <5% of detections were grouped into a separate category “Other” to allow for more robust comparisons of infrequent activities. To evaluate whether the probability of observing a given behavior differed between regions, we generated binary response variables (e.g., 1 = Investigating, 0 = otherwise) and fitted generalized linear models (GLMs) with a binomial error distribution and logit link. Region was included as the predictor variable.

To determine whether mink “investigatory” behavior varied with the time elapsed since the most recent otter observation, we analyzed event‐level detections using GLMMs. Models were fit with a binary response variable which defined whether investigatory behavior occurred per mink detection. Probability of investigatory behavior was modeled with a binomial error distribution and logit link. Time since last otter observation (hours) was included as a continuous predictor, modeled using a spline (df = 3) to allow for nonlinear attenuation of behavioral response over time. To avoid over‐interpretation of sparsely sampled time intervals, analyses were restricted to mink detections within 168 h (7 days) of the most recent otter observation. Month was included as a fixed effect, along with random intercepts for camera ID and date. Overall effects of time since otter presence were evaluated using Wald Type II χ^2^ tests.

The significance level for all statistical tests was set at α = 0.05. Model coefficients, test statistics, and associated *p*‐values are reported; effects with *p* < α were considered statistically significant. All statistical models and figures were generated in R version 4.4.

## RESULTS

### General results

The total number of trap nights in the expansion and established regions was 11,214 and 11,211 respectively. A combined 26,918 independent observations were made of 71 species during the course of the study. Otters and mink were the most commonly detected species (*n* = 6788 and 5711, respectively), though several rodent species were commonly observed along with passerines and shorebirds (Appendix S1: Table S2). A total of 107 and 29 instances of mink carrying food items occurred in the expansion and established areas, respectively (Appendix S1: Table S3). Fish made up 93% of mink prey items recorded within each study area, with rodents, amphibians, birds, and crustaceans accounting for similar proportions of the remainder of the food items. Of the identifiable fish, wrasses (*Labridae* spp.) were the most common in both the expansion (44%) and the established area (70%).

### Otter site occupancy and detection frequency

As expected, otters were active on all camera traps in the established region (*n* detections = 6634) and litters were present at all camera locations (*n* detections = 639). While otters were detected on 20 cameras (59%) in the expansion region throughout the study period, independent sightings were sporadic and equaled just 2% that found in the established area (*n* = 154). Mean daily site occupancy for otters in the expansion area was just 1% (SD = 2.2%), significantly less than the established area at 40% (SD = 10.9%) (Figure 2, $\hat{\beta }$ = 3.98, SE = 0.09, *z* = 45.5, *p* < 0.001).

**FIGURE 2:**
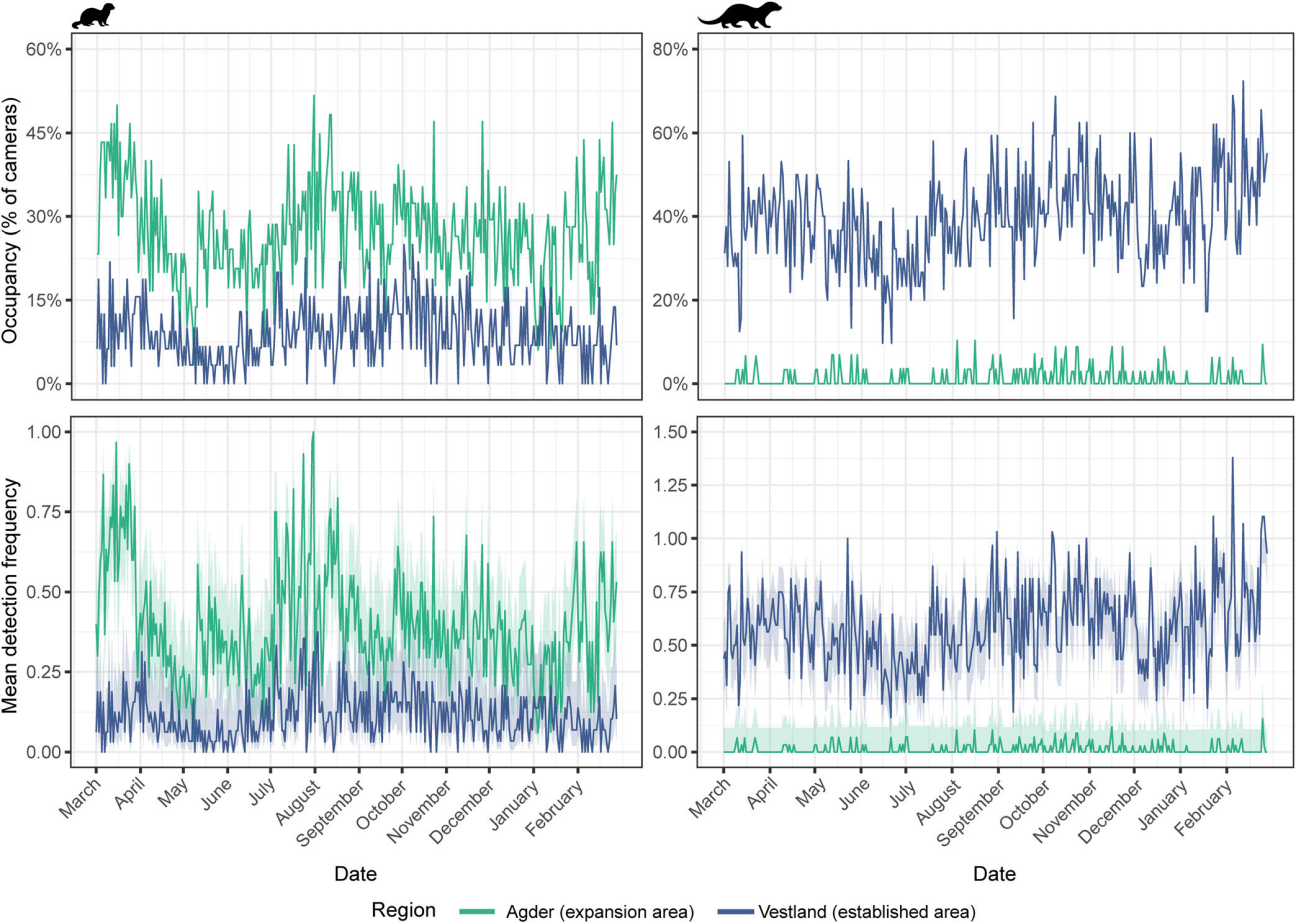
Proportion of camera locations occupied per day (top row) and mean detection frequency (bottom row) of mink (left column) and otter (right column) over the course of the study, split by study region. Shaded region of mean detection frequency indicates 95% CIs per day. Otter and mink silhouettes were created using generative software (OpenAI, ChatGPT).

In addition, mean detection frequency (Figure 2) was significantly less in the expansion area (0.01 detections/day) compared to the established region (0.59 detections/day, $\hat{\beta }$ = 4.27, SE = 0.40, *z* = 10.68, *p* < 0.001). Month had a significant impact on otter detection frequency in the established area (χ^2^ = 104.54, df = 11, *p* < 0.001), revealing seasonal changes in daily detection rates: lowest in June and July and peaking from September to October and again in January–February. There was no seasonal variation in otter activity in the expansion region, with low detection frequencies throughout the study period (Figure 2). Otter detection frequency was not impacted by amount of precipitation or temperature. However, wind conditions in the established region had a noticeable effect on detection frequency levels. After accounting for seasonal effects and repeated observations at cameras, detection frequency decreased with increasing wind speed (χ^2^ = 7.30, df = 1, *p* < 0.01). Based on model estimates, a 10 m/s increase in mean daily wind speed was associated with an approximate 38% reduction in otter detection frequency.

No otter litters were recorded in the expansion region, and when possible to determine, all individuals were male. Although otters were detected in the expansion region, these combined factors indicated a large difference in level of establishment between the study regions, with the expansion region's population likely being composed mostly of transient individuals.

### Mink site occupancy and detection frequency

The total number of mink detections in the expansion region was *n* = 4450, and *n* = 1261 in the established area. Over the course of the study, mink were captured on all cameras in the expansion region (*n* = 34), and on 31 out of 32 in the established area. Daily mink occupancy was significantly lower in the established region compared to the expansion area ($\hat{\beta }$ = −1.6, SE = 0.12, *z* = −12.96, *p* < 0.001), with mean daily occupancy values of 9% (SD = 5.3%) and 27% (SD = 8.7%), respectively. Month had a significant effect on daily site occupancy (χ^2^ = 141, df = 11 *p* < 0.001), which was highest in March (~37%) and August to October (~27%–33%), and decreased markedly in April–June and January within the expansion area. Site use in the established region was particularly low in May (4%) and then rose gradually with highest occupancy from July to November.

Similar to occupancy, mean daily detection frequency was 72% lower in the established area (0.11 ± 0.41) compared to the expansion region (0.39 ± 0.8 detections, $\hat{\beta }$ = −2.24, SE = 0.28, *z* = −8.11, *p* < 0.001). Seasonal variation in mink detection frequency was significant (χ^2^ = 229.13, df = 11, *p* < 0.001), though this effect was more pronounced in the expansion region. In the expansion area, daily detection frequency peaked in March (0.70 ± 0.13 detections per camera‐day) and declined significantly through April–June (Tukey‐adjusted *p* < 0.001). Detection frequency increased again during July and August, before declining from November through January. In the established area, mink detections remained comparatively low throughout the year, especially in May, which was significantly lower than most other months (Tukey‐adjusted *p* < 0.01), and there were modest increases during late winter (February). Inclusion of camera station significantly improved model fit in both the expansion region (χ^2^ = 1453.3, df = 1, *p* < 0.001, difference in AIC between models [ΔAIC] = 1451.3) and established area (χ^2^ = 647.6, df = 1, *p* < 0.001, ΔAIC = 645.6), indicating preference by mink for certain locations in each region. This appeared to be unrelated to local environmental characteristics (cover amount and cover type), which did not significantly improve model fit over the baseline model (χ^2^ = 1.02, df = 2, *p* = 0.59; ΔAIC = 3 and χ^2^ = 1.83, df = 2, *p* = 0.39; ΔAIC = 2, respectively).

Interestingly, mink detection frequency was positively associated with the recency of otter presence (χ^2^ = 22.27, df = 3, *p* < 0.001). The relationship was nonlinear, with predicted mink detection frequency decreasing as time elapsed since the previous otter detection. Model predictions indicated a 27% decrease in expected detection rate within 72 h after the last otter visitation, followed by a gradual decline until approximately 120 h since the last otter was present. Similar to otters, increasing wind speed was associated with reduced mink detections (χ^2^ = 28.21, df = 1, *p* < 0.001), equivalent to an estimated 32% decrease per 10 m/s increase in mean daily wind speed (95% CI: 22%–41%). Long‐term camera‐level otter detection rate did not significantly improve model fit after accounting for camera station as a random intercept, indicating that camera‐specific heterogeneity in baseline mink detection rates was already captured by the random‐effect structure.

### Rhythmic activity

Mink were more likely to be detected in the expansion region during the day (53%) compared to night (40%), while diurnal and nocturnal detections in the established area were evenly split at 46%, respectively (Figure 3). Crepuscular detections were also similar between the expansion region (7%) and established area (8%). Overall, no significant differences in activity period were found between the study areas (diurnal: *p* = 0.17, SE = 0.31, *z* = −1.37; nocturnal: *p* = 0.58, SE = 0.29, *z* = 0.54; crepuscular: *p* = 0.07, SE = 0.38, *z* = 1.89). Mink diel activity patterns in both regions varied in relation to photoperiod (Figure 3), with diurnal activity peaking in summer months, and waning in winter.

**FIGURE 3:**
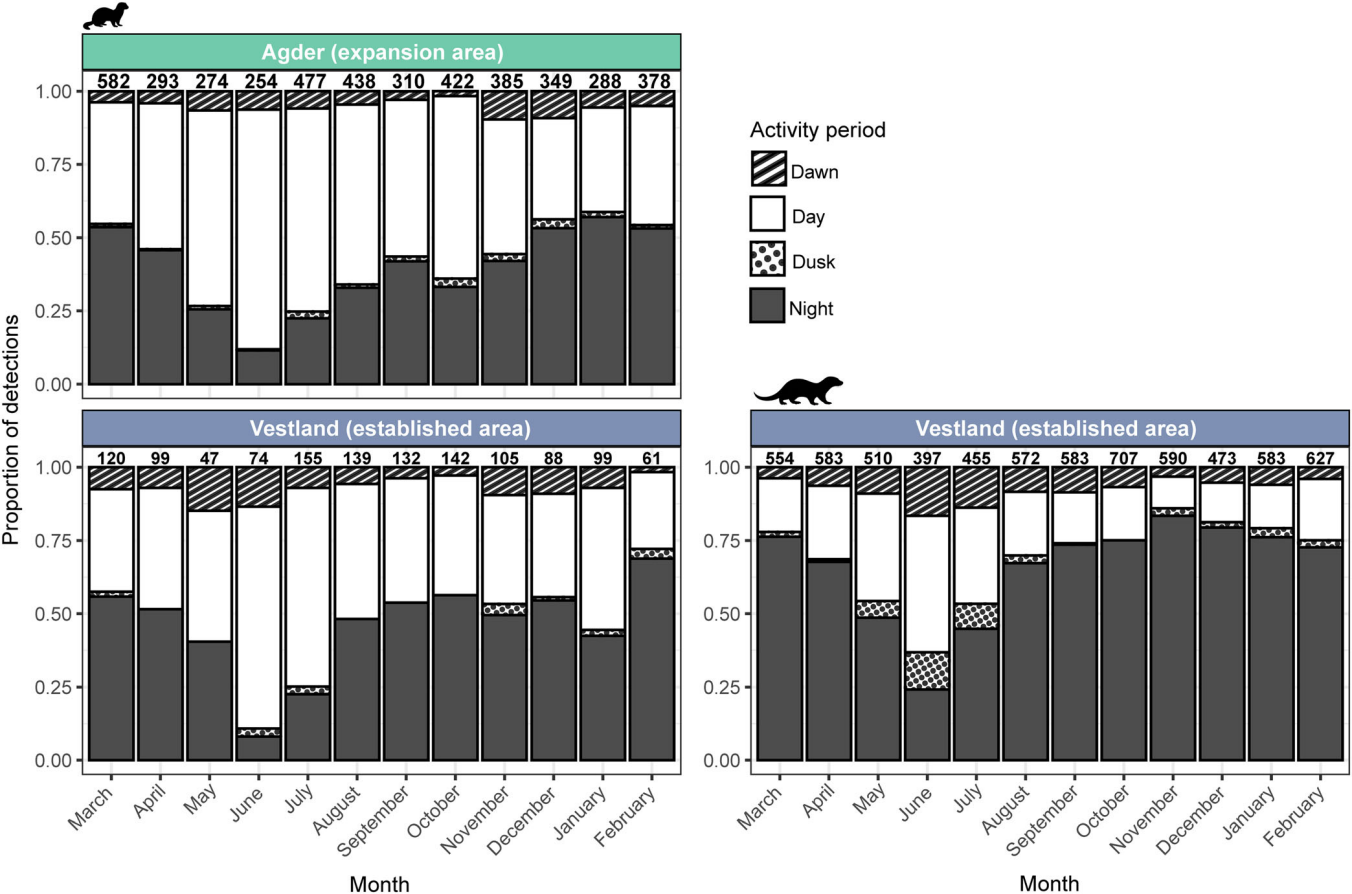
Proportion of mink (left) and otter (right) detections in each activity period, split by region. Otter rhythmic activity was omitted from the expansion region due to low sample sizes. Numbers over bars indicate sample size per month. Otter and mink silhouettes were created using generative software (OpenAI, ChatGPT).

Analyses of otter rhythmic activity were limited to the established region. Otter activity was mostly nocturnal (68%), and also related to changes in photoperiod throughout the year (Figure 3). Nocturnal activity peaked in November (84%) and was lowest in June (24%).

Mink were significantly more diurnal than otters within the established region (*p* < 0.001, SE = 0.08, *z* = 12.05). An odds ratio of 2.47 revealed that mink were more than twice as likely to be detected in the day compared to otters. This trend was repeated over all months of the study except for May and February (see Appendix S1: Table S4 for a detailed overview), at which times proportion of diurnal activity was roughly equal between the species (Figure 3).

### Behavior

Less than 5% of all mink detections in both regions included behaviors other than “traveling” and “investigating” (Figure 4). A binomial GLM indicated that the proportion of detections that included “investigating” behavior (21%) was significantly greater than in the expansion area (14%, *p* < 0.001, SE = 0.07, *z* = 6.53). In addition, time since the last otter observation at a camera trap had a significant impact on the probability of mink displaying “investigatory” behavior following the otter visit (χ^2^ = 8.9, df = 3 *p* = 0.03). Probability of mink displaying investigatory behavior within an hour of the last otter visit was 25% and dropped to 15% after 24 h, thereafter remaining stable (Figure 4).

**FIGURE 4:**
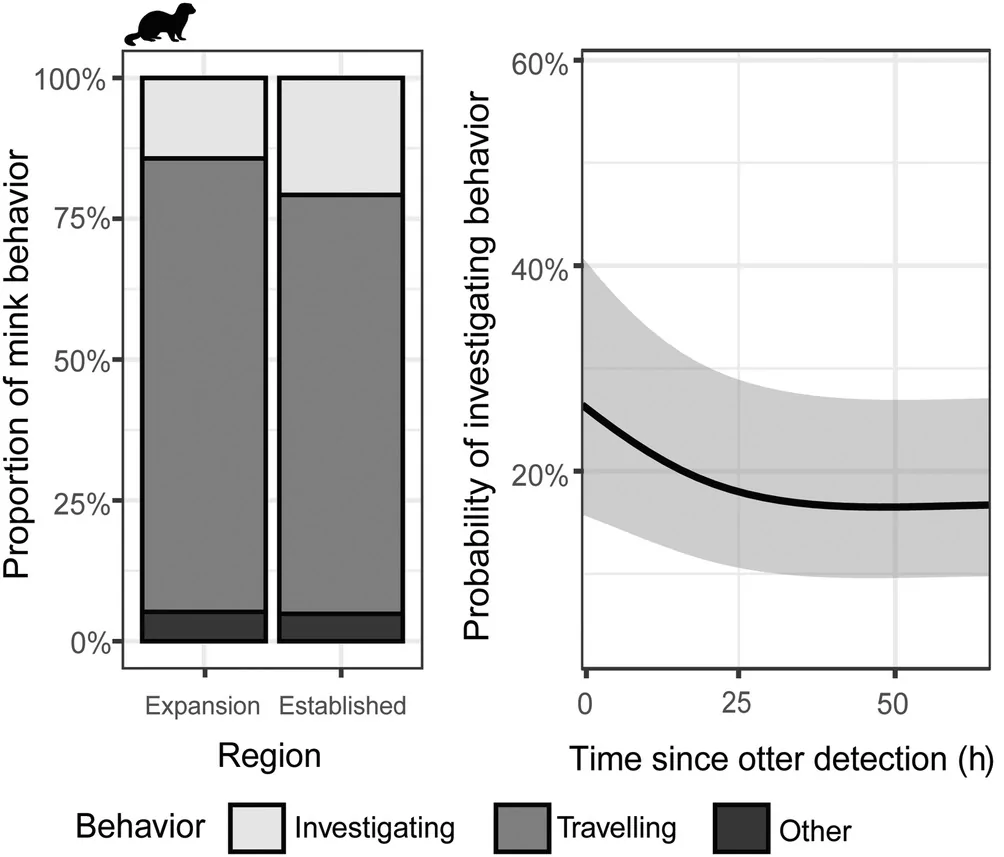
The proportion of mink detections for each behavior, split by region (left) and probability of mink investigatory behavior following time since last otter detection in the established area (right). Otter and mink silhouettes were created using generative software (OpenAI, ChatGPT).

## DISCUSSION

Meso‐predator site occupancy and density is often inversely correlated with that of apex predators (Jiménez et al., 2019; Newsome et al., 2017; Ritchie & Johnson, 2009), although individual cases may vary (van Schaik et al., 2025). Our results are consistent with this broader pattern: mink naïve occupancy and detection frequency were lower in the region with an established otter population than in the region where otter detections were sparse. This pattern is generally comparable to a previous study by Bonesi and Macdonald (2004c), who reported a significant drop in mink‐occupied sites in a river system following an intentional release of otters.

In some cases, competitive inferiors will alter habitat use in an effort to reduce overlap with dominant species (Davies et al., 2021; Dröge et al., 2017; Marneweck et al., 2019). We cannot confirm that mink have not shifted activity to areas inland of the coastline in an effort to reduce spatial overlap with otters, resulting in lower detection rates in the established region. However, we believe this is unlikely for several reasons. Previous radio‐tracking studies in freshwater systems indicate that mink did not change habitat use in the presence of otters when alternative habitat types were suboptimal (Harrington, Harrington, Yamaguchi, et al., 2009). Here, areas inland of the coastline in the fjord systems generally ascend abruptly to boreal coastal forest and alpine habitat, which offers lower prey diversity compared to coastlines. Mink densities are inherently tied to resource availability (Dunstone, 1993); hence, coastal areas with greater abundance of prey species generally boast denser mink populations than freshwater systems (Bonesi, 1996; Haan & Halbrook, 2015; Yamaguchi & MacDonald, 2003). While terrestrial prey species like rodents may potentially be seasonally abundant inland, these habitats include dietary competitors like pine martens and stoats (Breault et al., 2024). In addition, they host a dominant spatial competitor, the red fox, which may be particularly important as fox densities over time appear to be inversely correlated with that of mink (Carlsson et al., 2010). Relocation to areas inland of the coast would therefore not necessarily lead to a reduction of competition for food or space.

In addition to competition for space, past studies also show that otters and mink exhibit dietary competition, and in some cases, mink appear to alter dietary composition where there is overlap (Bueno, 1996; Clode & Macdonald, 1995; Harper et al., 2020). While not the main focus of our study, and with an admittedly low sample size in the established area (*n* = 29), mink did not seem to alter prey selection captured on camera and prey type and species composition overlapped heavily between study regions (Appendix S1: Table S3).

Opposite to our prediction, mink daily detection frequency was positively associated with time since last otter observation. However, this is probably related to daily weather conditions in which both species prefer to be active, rather than an affinity of mink to trail otters (e.g., for possible scavenging opportunities). High wind speeds (wave exposure) resulted in significant predicted reductions in daily detections of both species, with both preferring to be active in calm conditions. This overlap in preferred activity periods could theoretically lead to increased encounter rates between these species and is a possible explanation for reduced detections of mink in the established area.

Detection frequency of species on camera traps can vary widely between seasons (Kays et al., 2020), and observed activity patterns are often influenced by environmental parameters or changes in foraging strategy (Caravaggi et al., 2018; Ikeda et al., 2016; Tomita, 2021). Here, the pronounced swings in mink detections throughout the year in both study regions reflect known demographic patterns related to reproduction, of which a thorough overview can be found in Dunstone (1993), which provided a useful framework for interpreting seasonal variation in detections. Detections peaked in both regions in February and March, corresponding to the mink rut, at which time males are actively searching for females. This was followed by a lull in daily detections from April to June, wherein females restrict their activity close to natal dens. July and August saw another peak in detections, when the presence of kits in the ecosystem inflate detection frequencies. The kit dispersal period (September–October) maintains high activity levels, after which detections stabilize from November to January, the period in which the population density is considered “stable.”

Previous studies have shown that mink may switch from nocturnal to diurnal activity when sympatric with otters to avoid competition (Harrington, Harrington, Yamaguchi, et al., 2009). This is a common phenomenon among sympatric predators (Dröge et al., 2017; Finnegan et al., 2021) and within mustelid assemblages (Kupferman et al., 2021), whereby activity period of the competitive inferior defers to the dominant competitor. However, necessity of diel activity partitioning is usually context specific and can vary by location (Cozzi et al., 2012; Sévêque et al., 2022). Here, we found that mink were significantly less nocturnal than otters in the established region, but there was no difference in mink rhythmic activity patterns between the expansion and established regions. This finding suggests that current mink rhythmic patterns in the established region are sufficiently different from that of the otter to negate large‐scale activity pattern changes and avoid otter encounters. Timing of activity was related to photoperiod, not otter presence.

We found that mink behavior consists of more “investigative” behavior where they exist together with otters, and this was directly related to the amount of time elapsed since recording of the last otter visitation. Investigation of odors is hypothesized to result from a need to gain information on the donor (Hurst et al., 1997). Age of scent can be inferred from concentration of volatile compounds (Apps et al., 1989; Ferkin et al., 1995) which in turn enables the receiver to modulate their response based on the perceived likelihood of encountering the signaler (Roberts, 1998). Increased investigatory behavior following recent bouts of otter activity would make sense, as previous studies have shown confrontations to be violent, and favor the otter (Bonesi et al., 2000). We recorded one interaction between the study species over the course of this experiment, in which case a mink reversed direction and left the camera location upon detecting an otter which had appeared directly in its path. While this was an isolated event, it indicates that mink seem to avoid encounters with otters when possible.

Increased predator vigilance behavior is often negatively correlated with foraging efficiency (Creel et al., 2017), sometimes leading to behavioral changes (Zanette et al., 2003, 2011) or in extreme cases resulting in poorer body condition (Christianson & Creel, 2010). A past study found decreased body condition of mink following reestablishment of otters (Harrington, Harrington, Yamaguchi, et al., 2009). While decreased foraging opportunity was believed to be the most likely cause, it is unknown to what extent recency of otter activity influences stress levels or decisions on foraging patch selection by mink. Should preferred foraging areas be avoided due to risk of otter encounter, this could reduce mink body condition, with associated costs to pathogen resistance and reproduction in the long term.

## THE BROADER PERSPECTIVE

Apex predators are integral to maintaining food web structure (Estes et al., 2011; Ripple et al., 2014). Their top‐down control is a major driver in meso‐predator population structure, with associated benefits for smaller prey species (Ritchie & Johnson, 2009). In addition, they play an important role in cultivating a “landscape of fear” (Brown et al., 1999; Laundre et al., 2010) with cascading effects on herbivore movement patterns and population sizes, limiting overgrazing (Ripple et al., 2001; Ripple & Beschta, 2007; Terborgh et al., 2001). Here, we suggest that limiting invasive potential of competitors be added to the list of positive, top‐down mediated controls apex mammalian predators have on ecosystems.

Our results lend weight to past studies in Australia which indicate that apex predators like Dingoes (*Canis lupus dingo*) can negatively impact invasive red fox and feral cat (*Felis catus*) populations, with associated benefits for rodents (Feit et al., 2019; Gordon et al., 2015; Johnson & VanDerWal, 2009). Similarly, Tasmanian devils (*Sarcophilus harrisii*) limit feral cat densities (Cunningham et al., 2020), while wolf (*Canis lupus*) and lynx (*Lynx lynx*) occupancy is negatively correlated with that of invasive racoon dog (*Nyctereutes procyonoides*) in Finland (Selonen et al., 2022). Despite extensive documentation that shows removal of larger predators tends to facilitate “meso‐predator releases” (Prugh et al., 2009; Ritchie & Johnson, 2009), this effect is relatively poorly documented for native mammalian predators on invasive competitors. Indeed, large‐scale removal of many of Earth's apex predators, and continued persecution and conflict has limited opportunities to conduct studies where the return of native predators on invasive meso‐predators can be tested.

In Norway, the wolf, brown bear (*Ursus arctos*) and wolverine (*Gulo gulo*) populations are highly regulated, even in national parks. Here, livestock grazing interests are generally the main opponents to large‐scale reestablishment of these species (Jacobsen & Linnell, 2016). The otter currently faces similar conflict in salmon rivers from local fishing organizations, and in certain coastal regions where the collection of eider duck (*Somateria mollissima*) down is still an important cultural tradition (Hanssen et al., 2026). While it is illegal to kill otters in Norway, culling permits can be issued by the government on a case‐by‐case basis when economic loss can be proven. In addition, the accidental drowning of otters in recreational fishing gear is still widespread, especially in western Norway (Landa & Guidos, 2020). Results here suggest a removal of otters from coastal habitats could weaken a potential native‐predator constraint on invasive mink, negating any would‐be ecosystem benefits from the suppression or spatial exclusion of the invasive, at least on the local scales described here. As such, the culling of otters should be avoided when possible, and measures should be enacted to reduce bycatch of otters.

## CONCLUDING REMARKS AND RECOMMENDATIONS

Differences in site occupancy, detection frequency and behavioral responses found here all point to fundamental differences between local mink populations that live sympatrically with otters compared to those which are allopatric or have minimal spatial overlap. However, we refrain from making direct links between detection frequency and mink population density between regions. We were not able to individually identify mink based on the camera trap footage, which limited the use of mark–recapture methods which have been used in the past to determine density for animals with identifiable features (Duľa et al., 2021; Maputla et al., 2013). Other methods like random encounter models (REMs) have been developed as a tool to overcome this barrier (Rowcliffe et al., 2008), and have been implemented successfully to obtain density estimates for other mustelid species which cannot be individually identified (Lewis et al., 2025; Manzo et al., 2012). However, a key assumption of REMs is that cameras must be placed randomly within a species habitat range, or stratified to cover areas of expected high and low occupation (Rowcliffe et al., 2013). All of our cameras were placed close to the shoreline in what could be considered “optimal” mink habitat. Without a more detailed understanding of mink habitat use in fjord systems, density estimates could potentially overestimate density in both regions.

Obtaining data on mink habitat use could strengthen results found here by verifying spatial occupancy between regions, territory sizes, and use of the coastal strip compared to inland regions. GPS data have recently been used to transform raw REM density estimates to linear habitat density for African clawless otters (*Aonyx capensis*) and could be applicable for mink given similar data (Lewis et al., 2025). A follow‐up study to gather GPS data from mink and otters living in both study regions is therefore highly recommended. Similarly, GPS data on coastal‐living otters could be equally useful for determining spatial overlap between the competitors and estimating otter population levels in coastal regions of Norway.

This study adds to the growing evidence of how competition from a native competitor can lead to apparent suppression of an invasive competitor (Carlsson et al., 2010; Cunningham et al., 2020; Gordon et al., 2015; Selonen et al., 2022). While the mink population in Norway is likely to persist, the return of the otter offers a potential boon to control efforts, and nationwide otter reestablishment should be encouraged.

## CONFLICT OF INTEREST STATEMENT

The authors declare no conflicts of interest.

## Supporting information


Appendix S1.

## Data Availability

Data and code (Guidos et al., 2026) are available in Dryad at https://doi.org/10.5061/dryad.rfj6q57s4.
