# Supplementary material for: Reestablishment of native top‐predator suppresses invasive competitor in coastal Norway
Source: Ecology. 2026 Nov 13;107(11):e70597. doi: 10.1002/ecy.70597 (PMC13768727; doi:10.1002/ecy.70597)
Supplement: Supplementary file 1 [file ECY-107-e70597-s001.pdf]

Appendix S1

Reestablishment of native top-predator suppresses invasive competitor in coastal Norway

Steven Guidos, Øystein Varpe, Jiska van Dijk, Arild Landa

Ecology

Table S1. An overview of local habitat characteristics (cover amount and type) for all camera traps.

Region	Camera Station	Cover amount	Cover type
**Established area**	C1	Partial	Vegetation
**Established area**	C2	Partial	Rock
**Established area**	C3	None	None
**Established area**	C4	Partial	Rock
**Established area**	C5	Partial	Rock
**Established area**	C6	Total	Rock
**Established area**	C7	Partial	Vegetation
**Established area**	C8	Total	Rock
**Established area**	C9	None	None
**Established area**	C10	None	None
**Established area**	C11	None	None
**Established area**	C12	Partial	Rock
**Established area**	C13	Partial	Vegetation
**Established area**	C14	Partial	Rock
**Established area**	C15	None	None
**Established area**	C16	Partial	Rock
**Established area**	C17	None	None
**Established area**	C18	Total	Rock
**Established area**	C19	Total	Rock
**Established area**	C20	Partial	Rock
**Established area**	C21	Partial	Vegetation
**Established area**	C22	Partial	Rock
**Established area**	C23	Partial	Rock
**Established area**	C24	None	None
**Established area**	C25	Total	Rock
**Established area**	C26	Partial	Rock
**Established area**	C27	Partial	Rock
**Established area**	C28	Partial	Rock
**Established area**	C29	Partial	Rock
**Established area**	C30	Total	Rock
**Established area**	C31	Total	Rock
**Established area**	C32	Partial	Rock
**Established area**	C33	None	None
**Established area**	C34	Total	Rock
**Expansion area**	IA1	None	None
**Expansion area**	IA2	Partial	Rock
**Expansion area**	IA3	None	None
**Expansion area**	IA4	Total	Rock
**Expansion area**	IA5	Total	Vegetation
**Expansion area**	IA6	Partial	Rock
**Expansion area**	IA7	Partial	Vegetation
**Expansion area**	IA8	Total	Rock
**Expansion area**	IA9	Partial	Vegetation
**Expansion area**	IA10	None	None
**Expansion area**	IA11	Partial	Vegetation
**Expansion area**	IA12	Partial	Vegetation
**Expansion area**	IA13	None	None
**Expansion area**	IA14	None	None
**Expansion area**	IA15	Partial	Vegetation
**Expansion area**	IA16	Partial	Vegetation
**Expansion area**	IA17	Partial	Rock
**Expansion area**	IA18	Partial	Vegetation
**Expansion area**	IA19	Partial	Rock
**Expansion area**	IA20	None	None
**Expansion area**	IA21	Total	Vegetation
**Expansion area**	IA22	Partial	Vegetation
**Expansion area**	IA23	Partial	Vegetation
**Expansion area**	IA24	None	None
**Expansion area**	IA25	Total	Rock
**Expansion area**	IA26	Total	Rock
**Expansion area**	IA27	None	None
**Expansion area**	IA28	Total	Rock
**Expansion area**	IA29	Partial	Rock
**Expansion area**	IA30	Total	Rock
**Expansion area**	IA31	Partial	Vegetation
**Expansion area**	IA32	Total	Rock

Table S2. Standardized species detection counts during the study period along with the number of cameras each species occurred at, split by study region. Detections were standardized temporally, specified in the Methods section.

Species	Expansion Region Count	Established Region Count	Expansion Cameras Detected	Established Cameras Detected	Total Count
Accipiter gentilis	0	4	0	1	4
Acrocephalus palustris	1	1	1	1	2
Actitis hypoleucos	53	463	9	27	516
Anas platyrhynchos	6	7	3	5	13
Anser anser	0	31	0	3	31
Anthus pratensis	96	85	17	19	181
Anthus trivialis	4	0	1	0	4
Apodemus sylvaticus	2966	416	33	19	3382
Ardea cinerea	21	82	5	14	103
Bufo bufo	2	0	1	0	2
Canis lupus familiaris	0	4	0	3	4
Capra hircus	0	305	0	4	305
Capreolus capreolus	30	11	4	4	41
Cervus elaphus	0	166	0	11	166
Charadrius hiaticula	0	1	0	1	1
Columba palumbus	1	5	1	4	6
Corvus corax	1	6	1	5	7
Corvus cornix	26	229	4	17	255
Cyanistes caeruleus	7	3	4	2	10
Cygnus olor	3	0	1	0	3
Emberiza citrinella	1	0	1	0	1
Emberiza schoeniclus	0	2	0	1	2
Erithacus rubecula	299	272	29	21	571
Felis catus	19	20	6	8	39
Fringilla coelebs	8	63	3	14	71
Haematopus ostralegus	6	223	2	7	229
Haliaeetus albicilla	0	31	0	12	31
Homo sapiens	6	55	5	24	61
Larus argentatus	4	39	1	6	43
Larus canus	0	14	0	4	14
Larus fuscus	0	2	0	1	2
Larus marinus	15	29	3	2	44
Lepus timidus	2	0	1	0	2
Lutra lutra	154	6634	20	32	6788
Martes martes	75	11	20	6	86
Meles meles	21	0	7	0	21
Mergus merganser	2	5	1	2	7
Motacilla alba	40	138	10	13	178
Motacilla flava	25	6	8	3	31
Mustela erminea	11	8	4	5	19
Mustela nivalis	3	0	2	0	3
Myodes glareolus	159	21	16	3	180
Neogale vison	4450	1261	34	31	5711
Numenius arquata	0	2	0	1	2
Oenanthe oenanthe	12	1	3	1	13
Ovis aries	26	280	2	6	306
Parus major	39	8	17	6	47
Phalacrocorax carbo	12	5	1	2	17
Phylloscopus collybita	0	1	0	1	1
Picus canus	5	7	4	5	12
Picus viridis	7	0	3	0	7
Plecotus auritus	0	3	0	1	3
Pluvialis apricaria	0	3	0	1	3
Prunella modularis	104	6	12	4	110
Rattus norvegicus	0	2	0	2	2
Rodentia	12	6	5	3	18
Saxicola rubetra	0	1	0	1	1
Sciurus vulgaris	60	64	13	10	124
Scolopax rusticola	4	129	2	21	133
Sitta europaea	2	4	2	3	6
Somateria mollissima	15	17	1	2	32
Sorex araneus	41	21	12	6	62
Sorex minutus	3	0	2	0	3
Spinus spinus	9	1	3	1	10

Tringa ochropus	0	1	0	1	1
Troglodytes troglodytes	204	215	26	11	419
Turdus iliacus	5	7	4	6	12
Turdus merula	278	1243	28	30	1521
Turdus philomelos	52	300	18	17	352
Turdus pilaris	2	1	1	1	3
Vulpes vulpes	84	144	18	9	228

Table S2 cont.

Table S3. Detections of otter (*Lutra lutra*) and mink (*Neogale vison*) with prey species on camera, split by study region. Prey type is noted for all observations, and prey family, genus and species are noted where this was possible to determine.

Date	Region	Predator species	Prey type	Prey family	Prey genus	Prey species	Common name
**2023-03-06**	Established	Mink	Fish	Gadidae			
**2023-03-06**	Established	Mink	Fish				
**2023-03-06**	Established	Mink	Fish				
**2023-03-08**	Established	Mink	Fish				
**2023-03-10**	Established	Mink	Fish	Gadidae			
**2023-03-10**	Established	Mink	Fish				
**2023-03-30**	Established	Mink	Fish				
**2023-03-30**	Established	Mink	Fish				
**2023-04-06**	Established	Mink	Fish	Pleuronectidae	Microstomus	kitt	Lemon sole
**2023-04-06**	Established	Mink	Fish				
**2023-04-16**	Established	Mink	Fish				
**2023-04-28**	Established	Mink	Fish	Labridae			
**2023-05-10**	Established	Mink	Fish				
**2023-05-13**	Established	Mink	Fish	Labridae			
**2023-05-18**	Established	Mink	Fish	Labridae			
**2023-05-18**	Established	Mink	Fish	Labridae	Ctenolabrus	rupestris	Goldsinny wrasse
**2023-05-19**	Established	Mink	Fish	Labridae			
**2023-05-19**	Established	Mink	Fish				
**2023-05-21**	Established	Mink	Fish				
**2023-05-23**	Established	Mink	Fish				
**2023-05-23**	Established	Mink	Fish				
**2023-05-31**	Established	Mink	Fish	Pleuronectidae	Microstomus	kitt	Lemon sole
**2023-05-31**	Established	Mink	Rodent				
**2023-06-06**	Established	Mink	Fish				
**2023-06-07**	Established	Mink	Fish	Pleuronectidae	Microstomus	kitt	Lemon sole
**2023-06-08**	Established	Mink	Fish				
**2023-06-08**	Established	Mink	Fish				
**2023-06-10**	Established	Mink	Fish				
**2023-06-10**	Established	Mink	Fish				
**2023-06-10**	Established	Mink	Fish				
**2023-06-10**	Established	Mink	Fish				
**2023-06-11**	Established	Mink	Fish	Pleuronectidae	Microstomus	kitt	Lemon sole
**2023-06-11**	Established	Mink	Fish				
**2023-06-12**	Established	Mink	Fish				
**2023-06-16**	Established	Mink	Fish	Labridae	Ctenolabrus	rupestris	Goldsinny wrasse
**2023-06-16**	Established	Mink	Fish	Pleuronectidae	Microstomus	kitt	Lemon sole
**2023-06-17**	Established	Mink	Fish	Pleuronectidae	Microstomus	kitt	Lemon sole
**2023-06-24**	Established	Mink	Fish	Labridae	Ctenolabrus	rupestris	Goldsinny wrasse
**2023-06-24**	Established	Mink	Fish				
**2023-07-04**	Established	Mink	Fish				
**2023-07-05**	Established	Mink	Fish	Labridae			
**2023-07-05**	Established	Mink	Fish	Labridae			
**2023-07-05**	Established	Mink	Fish	Pleuronectidae	Microstomus	kitt	Lemon sole
**2023-07-05**	Established	Mink	Fish	Pleuronectidae	Microstomus	kitt	Lemon sole
**2023-07-05**	Established	Mink	Fish				
**2023-07-05**	Established	Mink	Fish				
**2023-07-06**	Established	Mink	Fish	Labridae			
**2023-07-07**	Established	Mink	Fish				
**2023-07-07**	Established	Mink	Fish				
**2023-07-07**	Established	Mink	Fish				
**2023-07-12**	Established	Mink	Fish				
**2023-07-19**	Established	Mink	Fish				
**2023-07-22**	Established	Mink	Rodent				
**2023-08-10**	Established	Mink	Fish	Labridae			
**2023-08-17**	Established	Mink	Fish				
**2023-08-30**	Established	Mink	Fish				
**2023-09-21**	Established	Mink	Fish				
**2023-10-01**	Established	Mink	Fish				
**2023-10-11**	Established	Mink	Crab				
**2023-10-31**	Established	Mink	Fish	Labridae			
**2023-11-01**	Established	Mink	Fish				
**2023-11-02**	Established	Mink	Fish	Cottidae			
**2023-11-02**	Established	Mink	Fish				
**2023-11-02**	Established	Mink	Amphibian				
**2023-11-05**	Established	Mink	Fish	Labridae	Symphodus	melops	Corkwing wrasse
**2023-11-08**	Established	Mink	Fish	Pleuronectidae	Microstomus	kitt	Lemon sole

**2023-11-10**	Established	Mink	Fish				
**2023-11-10**	Established	Mink	Fish				
**2023-11-13**	Established	Mink	Fish				
**2023-11-15**	Established	Mink	Rodent				
**2023-11-15**	Established	Mink	Fish				
**2023-11-17**	Established	Mink	Fish				
**2023-11-20**	Established	Mink	Fish	Labridae			
**2023-11-20**	Established	Mink	Fish	Labridae			
**2023-11-21**	Established	Mink	Fish				
**2023-11-24**	Established	Mink	Crab				
**2023-11-25**	Established	Mink	Rodent				
**2023-11-26**	Established	Mink	Fish				
**2023-11-27**	Established	Mink	Fish	Labridae			
**2023-11-28**	Established	Mink	Fish				
**2023-11-28**	Established	Mink	Fish				
**2023-11-29**	Established	Mink	Fish				
**2023-12-01**	Established	Mink	Fish	Cottidae			
**2023-12-02**	Established	Mink	Fish	Gadidae			
**2023-12-04**	Established	Mink	Fish				
**2023-12-06**	Established	Mink	Fish	Labridae			
**2023-12-08**	Established	Mink	Fish				
**2023-12-08**	Established	Mink	Fish				
**2023-12-22**	Established	Mink	Fish				
**2023-12-23**	Established	Mink	Fish				
**2023-12-24**	Established	Mink	Fish				
**2023-12-24**	Established	Mink	Fish				
**2023-12-25**	Established	Mink	Fish	Pleuronectidae			
**2023-12-25**	Established	Mink	Fish	Pleuronectidae	Microstomus	kitt	Lemon sole
**2023-12-31**	Established	Mink	Fish				
**2024-01-07**	Established	Mink	Fish				
**2024-01-14**	Established	Mink	Fish				
**2024-01-28**	Established	Mink	Fish	Pleuronectidae	Microstomus	kitt	Lemon sole
**2024-01-29**	Established	Mink	Fish				
**2024-01-30**	Established	Mink	Fish	Gadidae			
**2024-01-30**	Established	Mink	Fish	Pleuronectidae	Microstomus	kitt	Lemon sole
**2024-01-30**	Established	Mink	Fish	Pleuronectidae	Microstomus	kitt	Lemon sole
**2024-02-04**	Established	Mink	Fish				
**2024-02-05**	Established	Mink	Fish	Pleuronectidae	Microstomus	kitt	Lemon sole
**2024-02-09**	Established	Mink	Fish				
**2024-02-17**	Established	Mink	Fish	Pleuronectidae	Microstomus	kitt	Lemon sole
**2024-02-25**	Established	Mink	Fish				
**2023-03-13**	Expansion	Otter	Fish				
**2023-03-16**	Expansion	Otter	Fish	Cyclopteridae	Cyclopterus	lumpus	Lumpfish
**2023-04-04**	Expansion	Otter	Fish				
**2023-04-18**	Expansion	Otter	Fish	Labridae	Labrus	bergylta	Ballan wrasse
**2023-04-19**	Expansion	Mink	Fish	Labridae			
**2023-04-23**	Expansion	Mink	Fish				
**2023-05-03**	Expansion	Otter	Fish	Gadidae			
**2023-06-16**	Expansion	Mink	Fish				
**2023-06-16**	Expansion	Mink	Fish				
**2023-07-05**	Expansion	Mink	Amphibian				
**2023-07-09**	Expansion	Mink	Fish	Labridae			
**2023-07-11**	Expansion	Otter	Fish	Pleuronectidae			
**2023-07-24**	Expansion	Mink	Fish				
**2023-07-25**	Expansion	Mink	Fish	Labridae			
**2023-07-25**	Expansion	Mink	Fish	Labridae	Ctenolabrus	rupestris	Goldsinny wrasse
**2023-07-30**	Expansion	Mink	Bird	Laridae	Larus	argentatus	Herring gull
**2023-07-30**	Expansion	Otter	Fish				
**2023-08-02**	Expansion	Mink	Fish	Labridae			
**2023-08-10**	Expansion	Otter	Fish				
**2023-08-14**	Expansion	Otter	Fish	Anguillidae	Anguilla	anguilla	European eel
**2023-08-16**	Expansion	Otter	Fish				
**2023-08-18**	Expansion	Mink	Fish				
**2023-09-20**	Expansion	Mink	Fish				
**2023-09-21**	Expansion	Otter	Fish				
**2023-09-23**	Expansion	Otter	Fish				
**2023-09-25**	Expansion	Otter	Fish				
**2023-09-29**	Expansion	Otter	Fish				
**2023-10-10**	Expansion	Mink	Fish				
**2023-10-22**	Expansion	Otter	Fish	Gadidae			
**2023-10-23**	Expansion	Mink	Fish	Labridae			

Table S3 cont.

**2023-10-26**	Expansion	Otter	Fish	Gadidae			
**2023-11-06**	Expansion	Otter	Fish	Pleuronectidae			
**2023-11-07**	Expansion	Otter	Fish				
**2023-11-12**	Expansion	Otter	Fish	Pleuronectidae			
**2023-11-21**	Expansion	Otter	Fish	Gadidae			
**2023-11-23**	Expansion	Mink	Fish	Labridae			
**2023-12-10**	Expansion	Otter	Fish	Lotidae	Brosme	brosme	Cusk
**2023-12-11**	Expansion	Otter	Fish	Pleuronectidae	Pleuronectes	platessa	European plaice
**2023-12-11**	Expansion	Otter	Fish	Pleuronectidae			
**2023-12-27**	Expansion	Mink	Fish				
**2024-01-01**	Expansion	Mink	Fish				
**2024-01-04**	Expansion	Otter	Fish				
**2024-01-05**	Expansion	Otter	Fish	Anguillidae	Anguilla	anguilla	European eel
**2024-01-05**	Expansion	Mink	Fish				
**2024-01-06**	Expansion	Mink	Fish				
**2024-01-09**	Expansion	Otter	Fish	Lotidae	Brosme	brosme	Cusk
**2024-01-16**	Expansion	Mink	Fish				
**2024-01-20**	Expansion	Mink	Fish	Gadidae			
**2024-01-20**	Expansion	Mink	Fish	Gadidae			
**2024-01-23**	Expansion	Mink	Fish				
**2024-01-23**	Expansion	Mink	Fish				
**2024-02-12**	Expansion	Mink	Fish				
**2024-02-19**	Expansion	Mink	Fish				
**2024-02-25**	Expansion	Mink	Fish	Pleuronectidae	Microstomus	kitt	Lemon sole
**2024-02-27**	Expansion	Mink	Fish				

Table S3 cont.

Table S4. Test statistics concerning the comparison of otter and mink diurnal activity patterns within the Expansion region for each month of the study.

**Month and year**	***p*-value**	**Standard error (SE)**	**Z statistic**	**Odds ratio**
**March 2023**	$p < 0.01$	0.45	2.78	1.94
**April 2023**	$p < 0.01$	0.47	2.84	1.97
**May 2023**	$p = 0.19$	0.51	1.30	1.54
**June 2023**	$p < 0.001$	1.07	4.07	3.50
**July 2023**	$p < 0.001$	0.81	6.09	3.72
**August 2023**	$p < 0.01$	0.45	3.22	2.04
**September 2023**	$p < 0.001$	0.53	3.96	2.41
**October 2023**	$p < 0.001$	0.58	4.72	2.72
**November 2023**	$p < 0.001$	1.03	5.31	3.98
**December 2023**	$p < 0.01$	0.67	3.25	2.44
**January 2024**	$p < 0.001$	0.97	5.43	3.89
**February 2024**	$p = 0.79$	0.35	0.26	1.09
